# Application of an antibody chip for screening differentially expressed proteins during peach ripening and identification of a metabolon in the SAM cycle to generate a peach ethylene biosynthesis model

**DOI:** 10.1038/s41438-020-0249-9

**Published:** 2020-03-15

**Authors:** Wenfang Zeng, Liang Niu, Zhaohui Wang, Xiaobei Wang, Yan Wang, Lei Pan, Zhenhua Lu, Guochao Cui, Weining Weng, Mingqiao Wang, Xun Meng, Zhiqiang Wang

**Affiliations:** 1 Zhengzhou Fruit Research Institute, Chinese Academy of Agricultural Sciences, 450009 Zhengzhou, China; 2 Abmart, 200233 Shanghai, China; 3 Northwest University, 710127 Xi’an, China

**Keywords:** Plant development, Protein-protein interaction networks

## Abstract

Peach (*Prunus persica*) is a typical climacteric fruit that produces ethylene rapidly during ripening, and its fruit softens quickly. Stony hard peach cultivars, however, do not produce large amounts of ethylene, and the fruit remains firm until fully ripe, thus differing from melting flesh peach cultivars. To identify the key proteins involved in peach fruit ripening, an antibody-based proteomic analysis was conducted. A mega-monoclonal antibody (mAb) library was generated and arrayed on a chip (mAbArray) at a high density, covering ~4950 different proteins of peach. Through the screening of peach fruit proteins with the mAbArray chip, differentially expressed proteins recognized by 1587 mAbs were identified, and 33 corresponding antigens were ultimately identified by immunoprecipitation and mass spectrometry. These proteins included not only important enzymes involved in ethylene biosynthesis, such as ACO1, SAHH, SAMS, and MetE, but also novel factors such as NUDT2. Furthermore, protein–protein interaction analysis identified a metabolon containing SAHH and MetE. By combining the antibody-based proteomic data with the transcriptomic and metabolic data, a mathematical model of ethylene biosynthesis in peach was constructed. Simulation results showed that MetE is an important regulator during peach ripening, partially through interaction with SAHH.

## Introduction

Peach (*Prunus persica* L.), which belongs to the Rosaceae family, is an important fruit tree species worldwide. According to their fruit texture and firmness, peach cultivars are classified into one of three groups: the melting flesh (MF), nonmelting flesh (NMF), and stony hard (SH) types^1,2^. The fruit of MF peach cultivars is soft and juicy when fully ripe, whereas the flesh of SH cultivar peaches remains firm after harvest. In contrast, NMF fruit gradually softens when overripe but never melts^1^. Fruit firmness is a critical consideration for efficient harvesting, handling, marketing, and storage^3^.

Ethylene is the major trigger and coordinator of fruit ripening and subsequent metabolic changes, which have a direct impact on fruit quality^4,5^. Peach is a typical climacteric fruit whose respiration rate increases concomitantly with a spike in ethylene biosynthesis during the ripening process^6^. The committed steps in ethylene synthesis are catalyzed by two enzymes: 1-aminocyclopropane-1-carboxylic acid (ACC) synthase (ACS) and ACC oxidase (ACO), whose abundance is regulated at the transcriptional level. The induction of *ACS1* is responsible for climacteric ethylene production^6^. The fruit of MF peach cultivars produces large amounts of ethylene via system-2 ethylene production during the fruit-ripening stage, which results in rapid fruit softening. System-2 ethylene production is caused by the high induction of *PpACS1* at the late-ripening stage. In contrast, the fruit of SH peach cultivars only produces basal levels of ethylene due to a low expression level of *PpACS1*^7,8^.

It was found that high levels of auxin (IAA) induce *PpACS1* expression during peach ripening^9,10^, which is unique to peach, as *ACS1* expression is independent of IAA in the ripening of other fruits such as tomato and grape^11,12^. Therefore, the potential regulatory factors and specific regulatory network of ethylene production in peach remain to be characterized. Genes related to fruit ripening were identified by transcriptomic and genetic analyses during the sequencing of the peach genome^12,13^. These genes mainly encode proteins related to ethylene synthesis, including transcription factors, and enzymes involved in cell wall reconstruction and isoprenoid biosynthetic pathways^10^. A comparison of peach mesocarp proteomes at the climacteric transition stage by two-dimensional gel electrophoresis identified 53 differentially expressed proteins^14^, including enzymes related to ethylene metabolism [e.g., ACO1, S-adenosylmethionine synthase (SAMS), and β-cyanoalanine synthase], carbohydrate import activity (e.g., sucrose synthase and α-amylase), and scavenging of reactive oxygen species.

MS/antibody-based proteome-level investigations of peach are relatively rare. Despite the considerable utility of MS for the separation and identification of proteins, a lack of appropriate antibodies has hampered the validation of protein expression profiles and the results of functional studies. Antibody-based proteomic strategies have provided extensive supporting data for mass spectrometry (MS)-based proteogenomic research. However, the generation of various antibodies is time consuming and costly. Furthermore, the large-scale production of antibodies is difficult to reproduce, especially in nonmodel species. An antibody library that targets a complete proteome would be ideal for antibody-based investigations of specific organisms.

The generation and application of a monoclonal antibody (mAb) library was first reported by Fujita et al^15^. *Drosophila melanogaster* nervous system proteins were used as antigens to generate 148 mAbs, which were then used in immunohistochemical assays^15^. Different mAb libraries containing 100–1000 mAbs were subsequently generated for antigens from various sources, including human liver mitochondrial proteins^16^, plasma membrane proteins from lung cancer patients^17,18^, soluble proteins from bamboo shoots^19^ and proteins from *Arabidopsis thaliana* flowers^20^. Many of the antigens of interest were identified by MS following an immunoprecipitation (IP) step. Antibody libraries^17,18^ can be used in combination with microarrays for high-throughput screening.

In this study, we performed large-scale screening based on a mAb library to understand the protein changes in peach fruit-ripening stages. A total of 42 proteins were identified by using the mAbs and further confirmed by Western blot analysis. Notably, Methionine synthase (MetE) and S-adenosylhomocysteine hydrolase (SAHH), which form an interaction complex, are involved in the fruit-ripening process. By combining transcriptomic, proteomic, and metabolic results, a systemic biological model was established, providing a specific model of ethylene biosynthesis regulation in peach fruit.

## Results

### Identification of differentially expressed proteins during peach ripening through mAb array screening

**Fig. 1:**
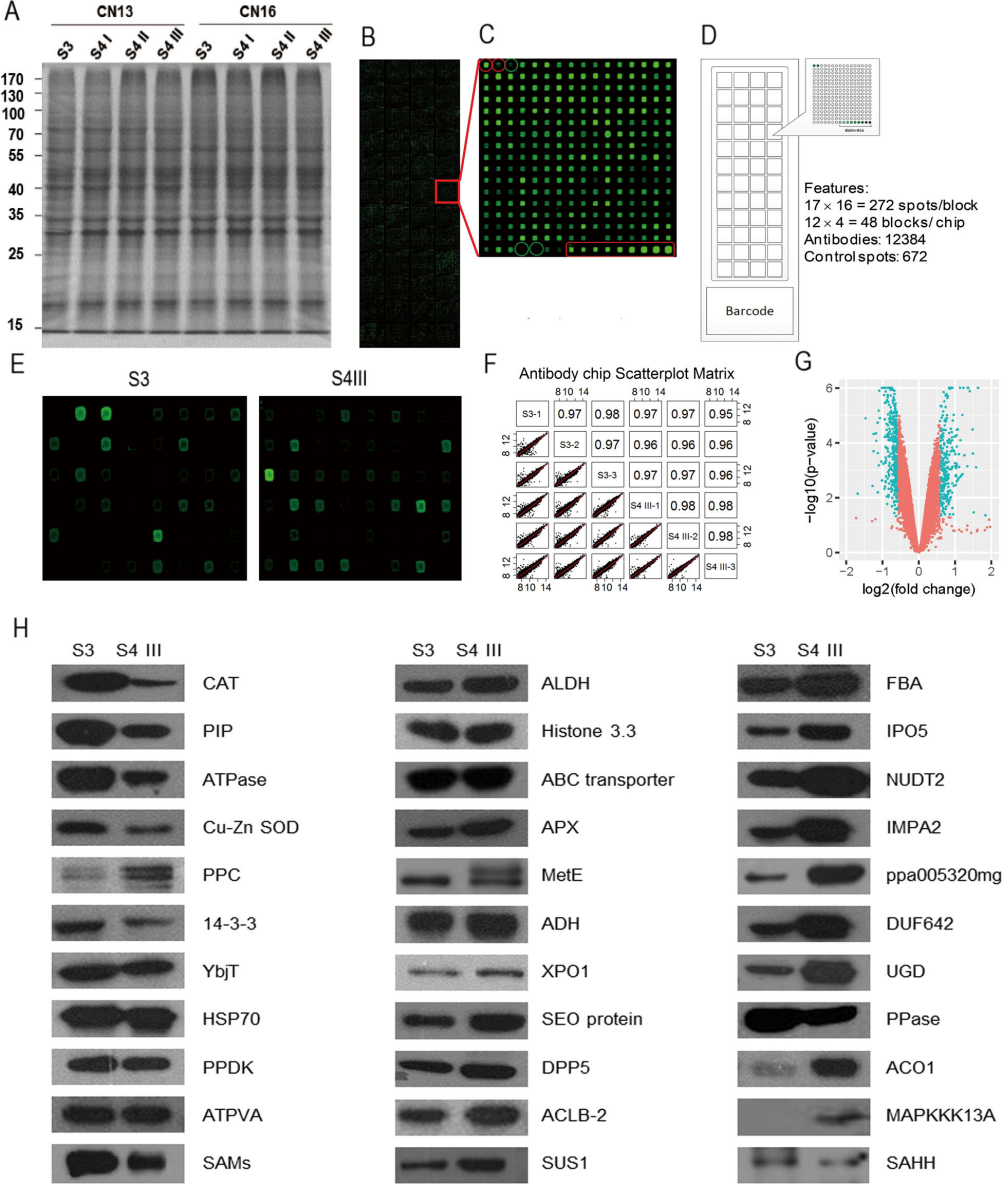
Construction of the peach mAb library and screening of differentially expressed proteins in ripening peach fruit with a mAbArray chip. **a** Peach protein antigens used to generate the mAb library. SDS-PAGE analysis of proteins extracted from peach fruit that were used as antigens for antibody library construction. Proteins were extracted from the mesocarp of the CN13 and CN16 cultivars at ripening stages S3, S4 I, S4 II, and S4 III. Twenty micrograms of protein was separated by SDS-PAGE and stained with Coomassie blue. **b–d** Quality of the antibody chip analyzed by hybridization. **b** Antibody chip after hybridization with Cy5-labeled goat anti-mouse IgG, and the Cy5 signal was scanned. **c** Magnified image of one block. The red circles and block indicate the positive controls (biotin-labeled BSA), and the green circles indicate the negative controls. **d** Diagram of mAb arrangement on the chip. **e** The peach mAbArray chip was used to screen for differentially expressed proteins in CN13 fruit between fruit-ripening stages S3 and S4 III. The differences in fluorescence intensities between peach fruit from stages S3 and S4 III within a small area of the chip are presented. **f** Correlation matrix of the replicated samples. **g** Volcano plot depicting the analysis of the differentially expressed proteins. The *p* value cut-off was 0.05 (y-axis), while the abundance fold-change cut-off was >1.5 or <−1.5 (x-axis). Significantly and nonsignificantly differentially expressed proteins are indicated in blue and red, respectively. **h** Western blot (WB) analysis of the differentially expressed proteins identified by LC-MS/MS. CN13 fruit samples harvested during stages S3 and S4 III were analyzed

Proteins from two types of peach (MF and SH cultivars) were used as antigens to generate a peach mAb library (Fig. 1a–d). Specifically, proteins were extracted from the mesocarps of fruit from the MF cultivar CN13 and the SH cultivar CN16 harvested between fruit-ripening stages S3 and S4 III (Fig. 1a). The generated peach mAb library contained 12,384 mAbs and was densely arrayed on a mAbArray chip (Fig. 1b–d). This mAb library was estimated to recognize 4950 different proteins (Fig. S1) and used to screen for differentially expressed proteins during fruit ripening. Protein expression in CN13 at the S3 and S4 III stages was compared via mAbArray chip analysis, and 1587 mAbs with significantly different signal intensities (*p* *<* 0.05) were identified (Fig. 1e–g). Western blot (WB) analyses indicated that 433 antibodies specifically recognized peach fruit proteins. Among the 433 mAbs, 100 mAbs that recognized proteins with different molecular weights were selected to further identify the target antigens. By immunoprecipitation (IP) and mass spectrometry (MS), 42 antigens corresponding to 33 different proteins were identified (Supplemental Table S1). The abundance of the 33 proteins was compared between S3 and S4 III in CN13 via WB (Fig. 1h). Dynamic changes in protein abundance were also characterized between CN13 and CN16 from S3 to S4 III during peach ripening (Fig. S2). WB analyses revealed 18 proteins, including CAT, PIP, PPase, 14-3-3, PPDK, SAMS, MetE, ADH, XPO1, SEO, ACLB-2, SUS1, IPO5, NUDT2, PPC, ACO1, MAPKKK13A, and SAHH proteins, whose abundance in CN13 and CN16 varied monotonically either between the two varieties or along with the ripening process (Table S1), suggesting their involvement in peach ripening.

### Proteins involved in ethylene biosynthesis exhibited different patterns during fruit ripening between the MF cultivar CN13 and the SH cultivar CN16

**Fig. 2:**
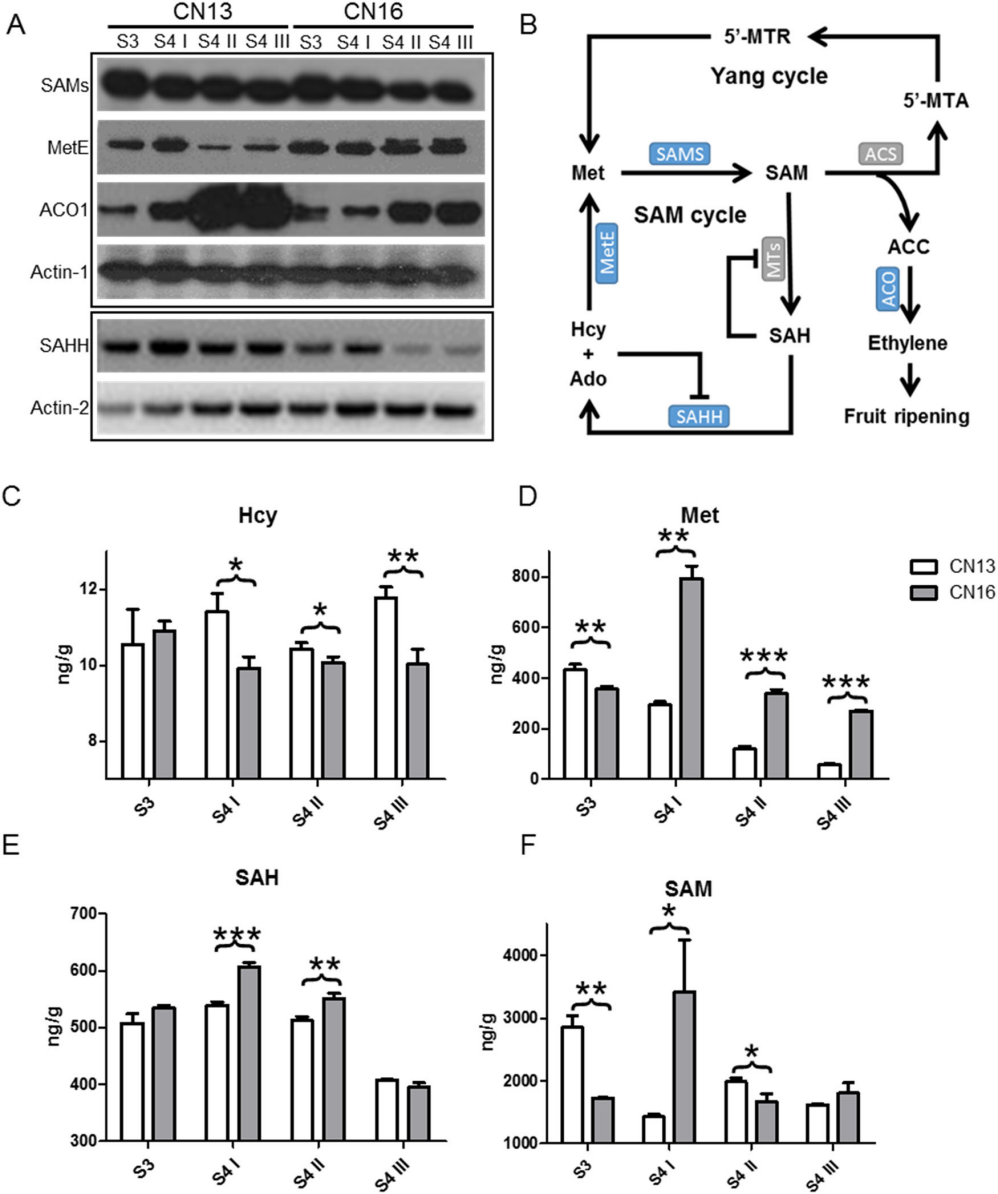
Analysis of enzyme abundance and metabolite changes during peach ripening. **a** WB analysis of SAMs, MetE, ACO1, and SAHH in CN13 and CN16 during ripening stages S3–S4 III. Actin-1 was used as a protein-loading control for the WB analyses of SAMs MetE and ACO1, and actin-2 was used as the control for SAHH. **b** Diagram of the recycling of methionine via the SAM and Yang cycles. The major enzymes and metabolites involved in sulfur group recycling through the SAM cycle and ethylene synthesis through the Yang cycle are indicated. Monoclonal antibodies recognizing the enzymes in blue were obtained in this study. **c–f** Measurement of metabolites in the SAM cycle during ripening stages in peach. The contents of **c** DL-homocysteine (Hcy), **d** l-methionine (Met), **e** S-(5′-adenosyl)-l-homocysteine (SAH), and **f** S-(5′-adenosyl)-l-methionine (SAM) were measured. Values are means ± SDs, *n* = 3. The significance of the differences was analyzed by Student’s *t*-test. ***p < 0.001; **p < 0.01; *p < 0.05

Special attention was paid to the proteins involved in the biosynthesis of ethylene. Although the abundance of ACO1 increased significantly in both CN13 and CN16 during the ripening stages, the increase in ACO1 in CN13 was more dramatic than that in CN16. However, the abundance of SAMS, which catalyzes the formation of SAM from methionine, remained nearly constant during ripening (Fig. 2a). In addition to these proteins directly involved in ethylene biosynthesis, MetE, which catalyzes methionine recycling from SAM and regulates the SAM distribution flux (Fig. 2b, ref. ^20^), was downregulated during the ripening process in CN13 but upregulated in CN16 at S4 II and S4 III. SAHH, which is also involved in SAM recycling, was upregulated at the S4I stage compared with the S3 stage and downregulated in CN16, but not CN13, at S4 II and S4 III (Fig. 2a).

To determine whether differential changes in MetE and SAHH in CN13 or CN16 during ripening result in different levels of metabolites in the SAM cycle, DL-homocysteine (Hcy), l-methionine (Met), S-(5′-adenosyl)-l-homocysteine (SAH), and S-(5′-adenosyl)-l-methionine (SAM) were quantified (Fig. 2b–f). It was shown that the level of Hcy was significantly higher in CN13 than in CN16 during the S4 stage (Fig. 2c). In contrast, Met and SAH contents were significantly higher in CN16 (Fig. 2d, e). The SAM content showed a significant difference between CN13 and CN16 at both the S3 and S4 I stages (Fig. 2f).

### ADP-ribose pyrophosphatase (NUDT2) exhibits a significantly higher protein level in CN13 than in CN16

We identified several proteins presenting a monotonically increasing pattern as the fruit matured, such as NUDT2, MAPKKK13A, IPO5, and PPC (Fig. S2). Notably, NUDT2 shows a positive correlation with ethylene production and is highly homologous to AtNUDT2. To compare oxidative damage between the MF and SH cultivars, O_2_^−^ contents at four stages were measured. Significant increases were observed during stage S3 to stage S4I in both CN13 and CN16 (Fig. S3).

### Analysis of protein–protein interactions (PPIs) among the proteins related to fruit ripening

**Fig. 3:**
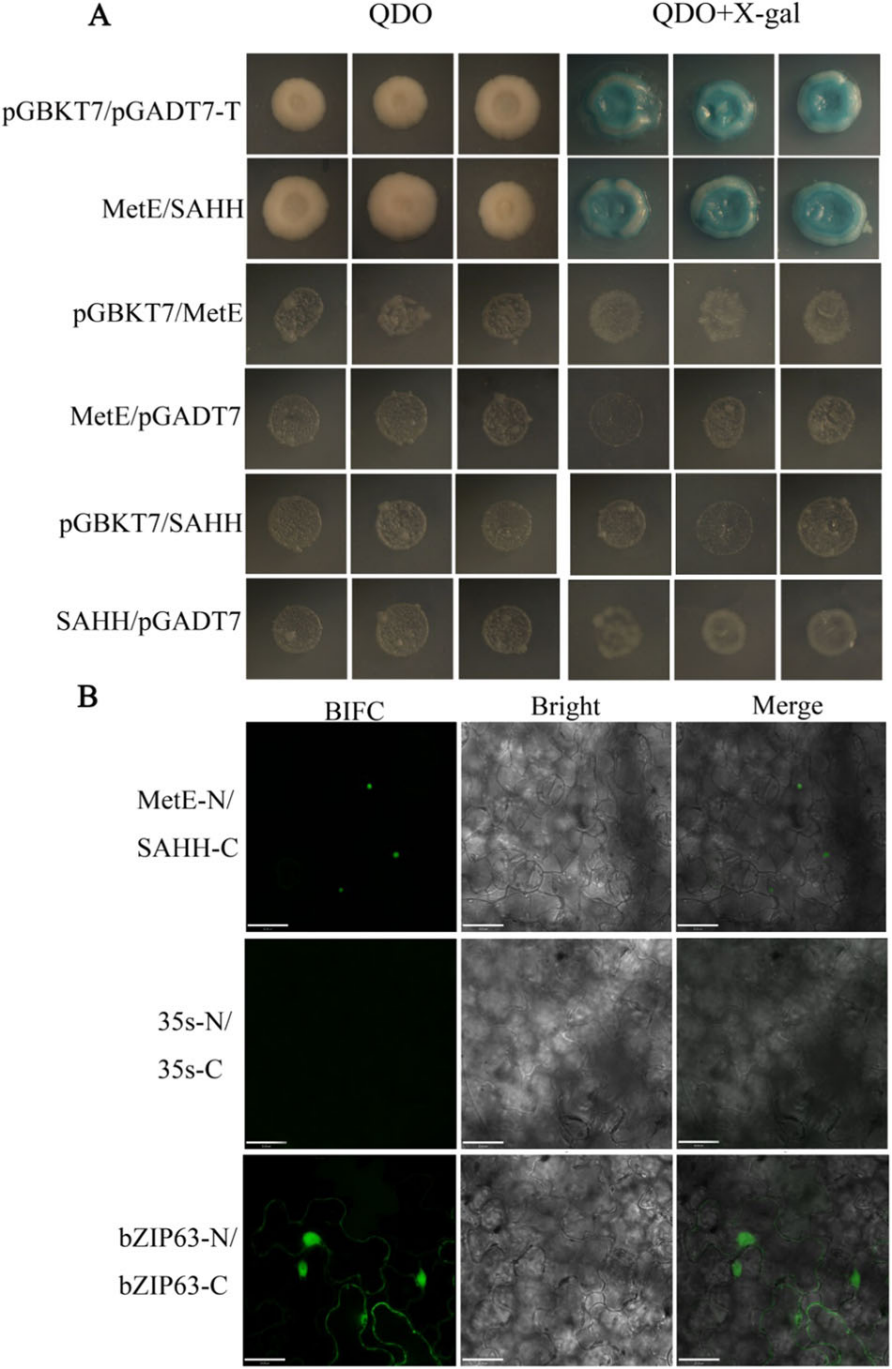
Detection of protein–protein interaction between MetE and SAHH. **a** Yeast two-hybrid analysis of the interaction between MetE and SAHH. Yeast transformants were grown on QDO medium (SD-Ade/-His/-Leu/-Trp) (left panel) or QDO medium supplemented with X gal (right panel). pGBKt7/pGADT7-T is a positive control. **b** BIFC analysis of the interaction between MetE and SAHH. The combined constructs (top to bottom) are PSPYNE-35S-MetE/PSPYCE-35S-SAHH, PSPYNE-35S/PSPYCE-35S (negative control), and PSPYNE-35S-bZIP63/PSPYCE-35S-bZIP63 (positive control). Bar = 32 μm

SAMS, SAHH, and MetE are enzymes involved in the SAM cycle that catalyze sequential reactions responsible for salvaging the SAM-bound sulfur group generated in methylation reactions catalyzed by methyltransferases^20^. To determine whether these enzymes may form a complex to facilitate the metabolic process, yeast two-hybrid (Y2H) and bimolecular fluorescence complementation (BIFC) assays were performed. As shown in Fig. 3, SAHH interacted with MetE.

In addition, co-IP assays coupled with MS were performed to explore the interacting proteins of SAMS, MetE, and ACO1. The interacting proteins were identified and classified into different functional categories (Fig. S4a). The putative interacting partners of ACO1 and MetE were enriched in proteins related to redox, storage, TCA, glycolysis, DNA synthesis, and amino acid metabolism (Fig. S4a). Among the proteins interacting with SAMS, proteins related to cell vesicle transport (e.g., clathrin, coatomer complex I (COPI) subunit, and GPI-anchored adhesion molecule) and G-protein signaling (RABs) were enriched (Fig. S4b). Interestingly, SAMS was found to interact with G proteins and vesicle transport proteins with known interactions, such as the interactions between SAMS and COPA1-Rab11A, COPE1-RABA2a and SRP72-RABB1c, suggesting the existence of putative protein interaction complexes. Furthermore, SAMS was found to interact with the cell wall-related enzyme that mediates fruit softening, endo-polygalacturonase (PG)^21^, and expansin A10 (EXPA10), a cell wall-loosening enzyme;^22^ these enzymes were highly expressed in MF fruit at the late maturation stage. An interaction between SAMS and MetE was found in reciprocal co-IP assays using anti-MetE and anti-SAMS mAbs, respectively (Fig. S4b). It was also predicted by the STRING database (Fig. S4b).

### Transcriptomic analysis of gene expression during fruit ripening in peach

**Fig. 4:**
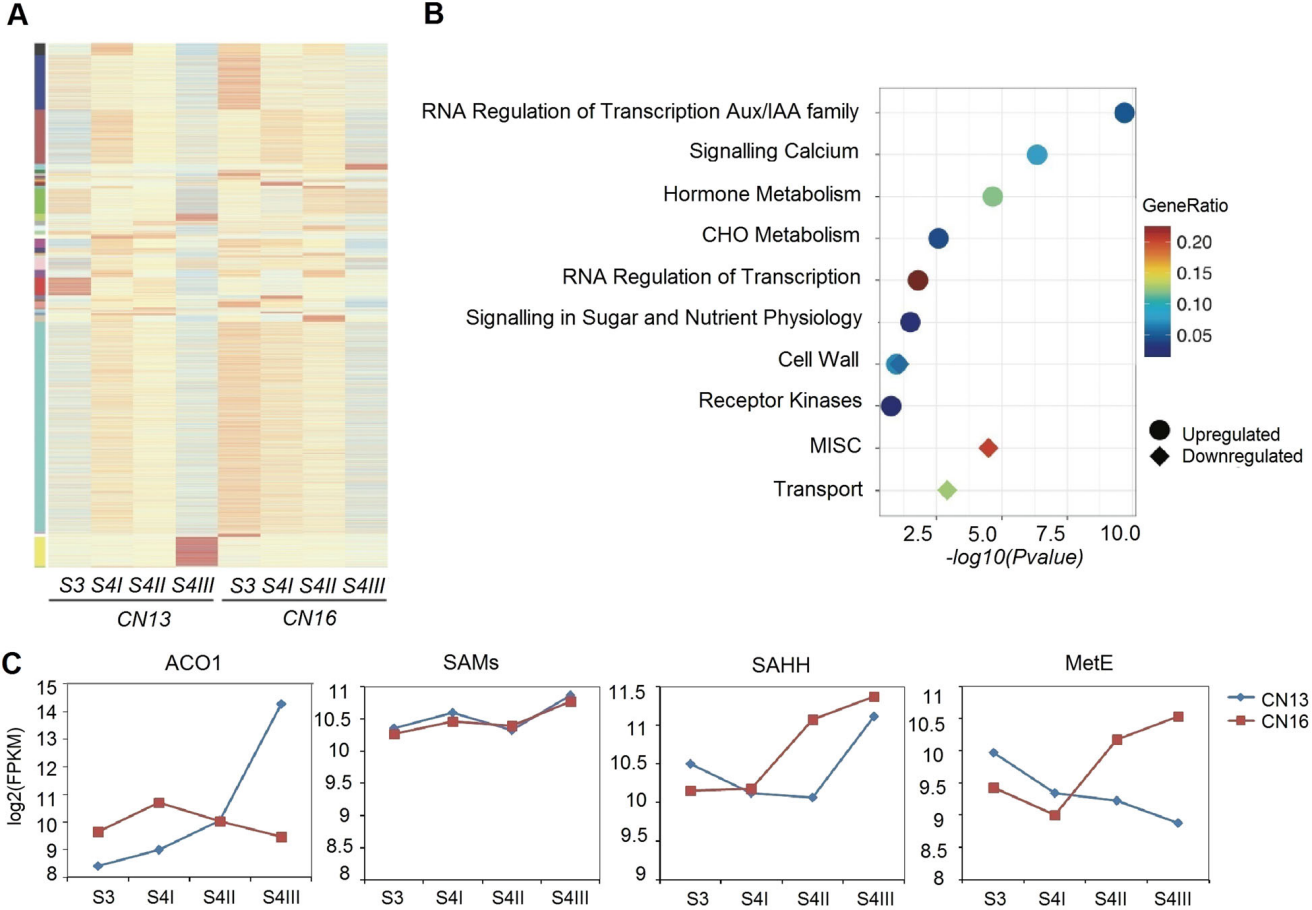
Functional analyses of differentially expressed genes during fruit ripening in peach. **a** Clustering analysis showing the coexpression modules identified by WGCNA. The different modules identified are color labeled. **b** Functional enrichment analysis of differentially expressed genes. The upregulated functions (filled circles) and downregulated (filled diamonds) functions are displayed with respect to their significance (*p* < 0.02). Different colors correspond to the ratio of genes in each category. **c** Gene expression during the ripening process in the melting flesh (MF) cultivar CN13 and the stony hard (SH) cultivar CN16

To capture the dynamic changes at the transcriptional level during fruit ripening, we performed RNA sequencing across the different stages of ripening in CN13 and CN16. The gene coexpression patterns associated with the different stages in CN13 and CN16 were analyzed by weighted gene coexpression network analysis (WGCNA)^23^ (Fig. 4a). Pathway enrichment analysis revealed eight upregulated and two downregulated categories of genes. The upregulated categories included genes involved in IAA-regulated transcription, calcium signaling, hormone metabolism, CHO metabolism, transcriptional regulation, sugar and nutrient signaling, and the cell wall (Fig. 4b).

Consistent with the trend indicated by the proteomic data, *ACO1* was among the most highly induced genes, showing an ~60-fold increase at stage S4 III in CN13, whereas it remained at a low level in CN16 (Fig. 4c). In contrast, *MetE* expression was decreased by approximately two-fold in CN13 cells (Fig. 4c). *SAHH* expression was induced earlier in CN16 than in CN13 during ripening stages and was ~2-fold higher at S4 III than S3 in both CN13 and CN16. *SAMS* expression did not change significantly throughout the ripening stages, and *SAHH* expression increased approximately 2-fold in both CN13 and CN16 (Fig. 4c).

### Construction of an integrative network related to ethylene production composed of key factors identified through multiomic analyses

**Fig. 5:**
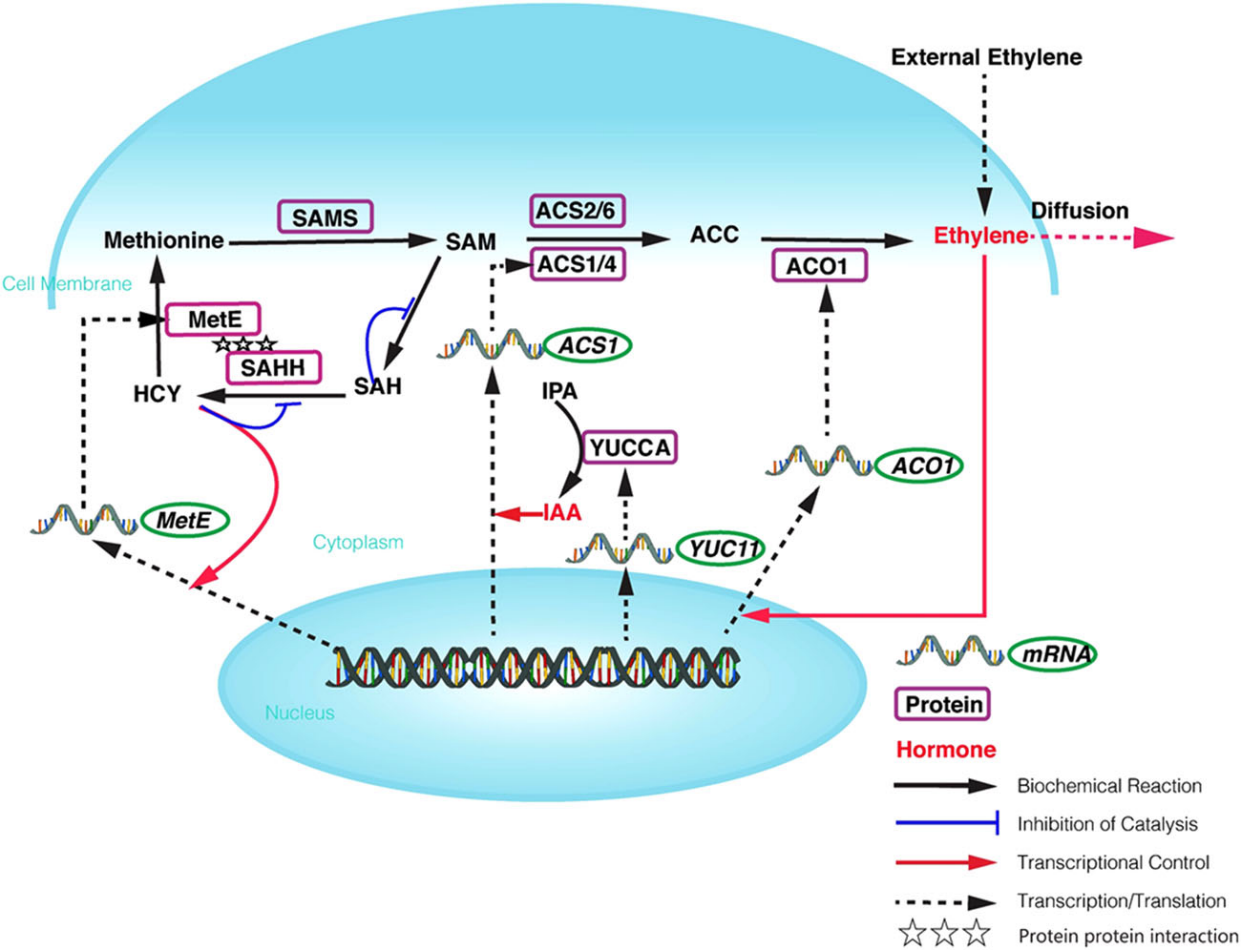
Biochemical network of the ethylene biosynthesis pathway during fruit ripening identified via proteomic analysis with the mAbArray

Key differentially expressed factors associated with ethylene biosynthesis during peach ripening were selected to construct a thorough model of ethylene production specifically during peach ripening. Starting from the four key proteins and their corresponding genes (SAMS, SAHH, MetE, and ACO1) (Figs. 2a and 4c), we filled in the necessary metabolites to form a complete metabolic network for modeling purposes (Fig. 5).

In addition to the metabolic reactions mentioned above, *ACS1* expression is induced by IAA, which is catalyzed by YUCCA from the indole-3-pyruvic acid (IPyA) pathway;^24,25^ the transcription of *MetE* has been reported to be linearly correlated with that of its substrate, Hcy^26^ (Fig. 5). In addition, ACO1 protein and mRNA levels both increased during the ripening process, suggesting a correlation between ethylene biosynthesis and *ACO1* expression during ripening stages^27^. The resulting network included not only ethylene production by SAM but also the feedback from the SAM cycle back to Met, which controls the SAM flux (Fig. 5). Furthermore, we experimentally identified the protein-protein interaction between MetE and SAHH both in vitro and in vivo, and the existence of this catalytic complex in the SAM cycle controls the efficiency of the SAM flux in the integrative network.

### Simulations with a kinetic model suggest key roles of both hormonal and transcriptional regulation during fruit ripening

We optimized the parameters via least squares estimation (detailed in the Methods). The levels of mRNA and protein and the measured metabolites were used as inputs for parameter estimation (Figs. S5 and S6). The enzymatic reactions of the core ethylene production pathway (from SAM to ethylene) are conserved across climacteric ripening species, and related parameters were adopted from Van de Poel et al^28^. This calibrated model works well in reproducing the dynamics of the aforementioned metabolic (Fig. S6), transcriptomic (Fig. S5) and proteomic measurements.

**Fig. 6:**
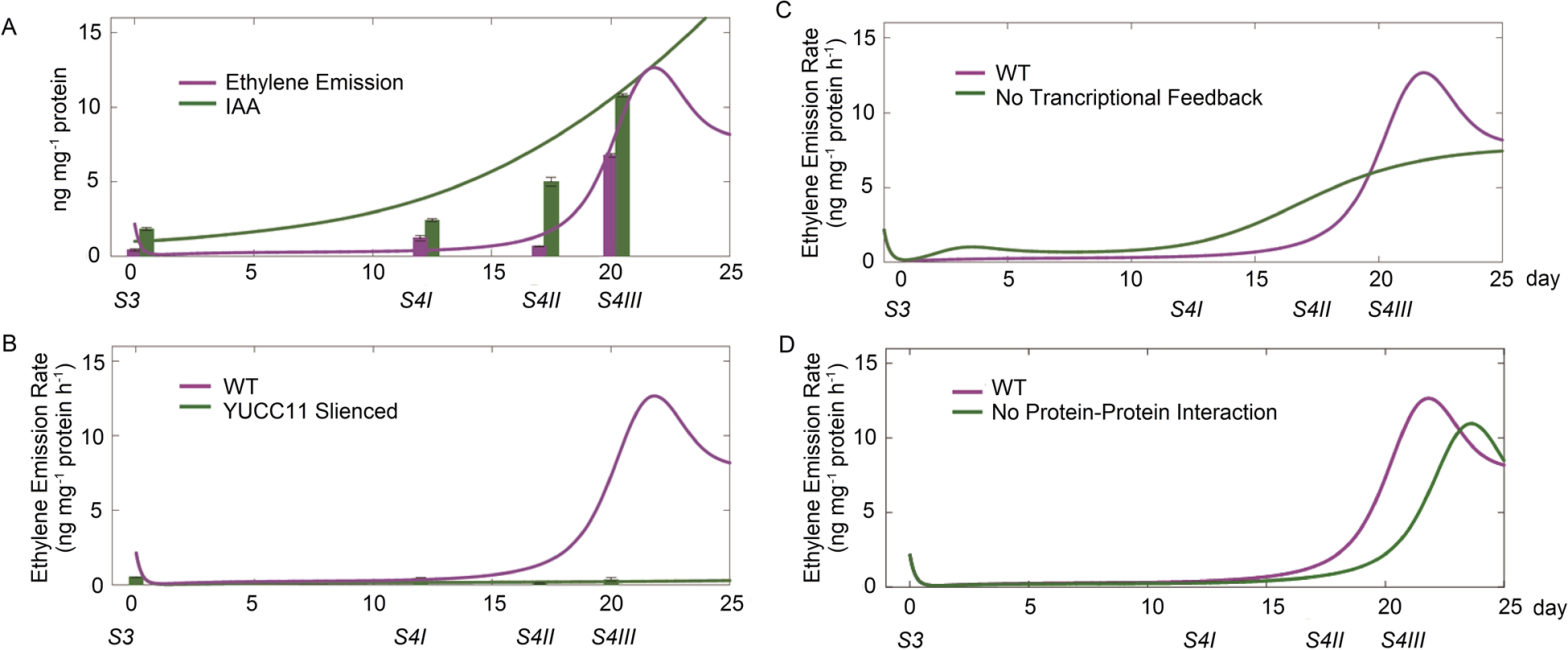
In silico dynamics of ethylene biosynthesis and emission in fruit under various conditions during fruit ripening. **a** In MF cultivars, IAA initiates ethylene biosynthesis, and the simulations were compared with experimental measurements (magenta bar: IAA concentration; green bar: ethylene emission rates of CN13). **b** The expression of *YUC11* in SH fruit during fruit ripening is silenced. The simulation of ethylene emission is compared with experimental measurements (green bar: ethylene emission rates of CN16). **c** In MF cultivars, the identified transcriptional feedback of *ACO1* is absent. **d** In MF cultivars, the identified protein–protein interaction between MetE and SAHH is absent

After the parameterization of this integrative model with transcriptomic, metabolic, and proteomic quantification results, we simulated the dynamics of ethylene and IAA synthesis during fruit ripening in CN13 peaches (Fig. 6a), and the concentrations of both hormones matched the experimental measurements, which are shown with bars at four different stages during fruit ripening. In addition to ethylene production during fruit ripening, we observed a decrease in the internal ethylene concentration and ethylene emission rate during the postclimacteric stages (Fig. 6a), which has also be observed in tomato^28^. This decrease in ethylene production resulted from the continuous accumulation of the ACS1 and ACO1 enzymes during fruit ripening (Fig. 6a), which rapidly depleted ethylene precursors.

We then checked the effect of IAA on ethylene synthesis with our model. As shown in Fig. 6a, b, the IAA concentration increased prior to a sharp increase in ethylene emission in CN13, while the silencing of *YUC11* in CN16 turned off IAA production and ethylene generation, which was consistent with the experimental data^24,25^. IAA regulates ethylene production through the induction of Type-II ACS genes. These results together strongly confirmed the determinant role of IAA in the regulation of ethylene biosynthesis in peach fruit ripening.

An important feature of this integrative model is the incorporation of direct feedback control as proposed^27^ and validated from our data. To evaluate the significance of this transcriptional feedback regulation of *ACO1*, we predicted the dynamics of ethylene production without transcriptional regulation, which indicated that ethylene synthesis would be decreased and delayed compared to the control (Fig. 6c). This feedback control acts as a signal amplifier and is responsible for the exponential increase in *ACO1* expression (Fig. S5c), protein synthesis, and ethylene production observed during climacteric fruit ripening.

Additionally, we predicted the effect of protein–protein interactions on ethylene synthesis. The production of Met from Hcy is catalyzed by MetE, and the synthesis of Hcy is regulated by SAHH from SAH. The interaction between MetE and SAHH aids in the transportation of metabolites between the two catalytic machines. We simulated a situation in which the SAHH-MetE interaction is absent and metabolic channeling does not exist. The results showed delayed ethylene emission and maturation compared to the normal MF cultivar (Fig. 6d). “Late maturation” results from the slow recycling of Met from SAH, while the existence of “metabolic channeling” allows the rapid recycling of Met and the production of ethylene thereafter, which helps to balance the metabolites between the Yang cycle and the SAM cycle of biochemical reactions.

## Discussion

The recent technological advances in proteomics and transcriptomics have generated enormous amounts of data; however, the application of these data to reveal regulatory mechanisms and understand biological problems at the systematic level remains a challenge^29^. By combining mAbArray-based proteomics and RNA-seq-based transcriptomics with a systems biology approach, we obtained functional networks based on measured differences and a parameterized mathematic model. We then applied this combinatorial approach to the regulation of ethylene biosynthesis in peach ripening.

### Convenience of mAb Mega-Chips in the quantification of proteomic differences

Peach has become the reference species for *Prunus*;^12^ however, the lack of a genetic modification system for peach remains the major obstacle to the functional validation of its genes/proteins. In this study, we successfully generated the first mega mAb library for peach fruit, from which ~1733 mAbs could be used in WB and IP experiments (Fig. S1). The application of microarray technology further increases the utility of mAb libraries by increasing the screening efficiency of mAbs for specific targets. The application of the mAbArray chip for studying the peach fruit ripening process led to the identification of differentially expressed proteins by 1587 mAbs. In addition, the library could be useful for the identification of marker mAbs for specific tissues, subcellular organelles, or developmental stages and for immunohistochemistry assays^30^. Therefore, the antibody library-based strategy provides an efficient way to obtain specific antibodies for nonmodel plants and a convenient screening and validation platform for functional proteomics. In our study, several proteins involved in ethylene biosynthesis, such as ACO1, SAMS, SAHH, and MetE, were differentially detected in MF and SH cultivars, indicating that mAb-based analysis can be used to investigate the changes in protein levels during peach ripening.

### A putative methionine metabolon involving SAHH and MetE

By constructing and screening the peach antibody library, we identified not only the key proteins involved in the ripening process but also the corresponding interactive protein networks via Y2H or BIFC assays and antibody-based co-IP assays. Metabolons have been proposed for a variety of specialized metabolic pathways in plants, including polyamine^31^, isoprenoid^32^, alkaloid^33^, and phenylpropanoid^34^ synthesis, although metabolic channeling has only been observed in three metabolons involved in glycolysis^35^, cyanogenic glucoside biosynthetic pathway^36^, and the TCA cycle^37^. We propose another putative metabolon involved in methionine recycling and ethylene synthesis with SAHH and MetE as the major components. SAM is highly important for ethylene synthesis; however, SAMS protein abundance remained constant, and MetE was reduced in MF peaches. On the other hand, Met and SAM contents decreased during peach ripening in CN13, while Met and SAM contents reached a peak in the CN16 cultivar and were depleted thereafter (Fig. S6), which could be related to the upregulation of MetE at the late-ripening stage (Figs. 2e and 5c). In addition, when SAM is not utilized for ethylene production, the protein–protein interaction between MetE and SAHH may contribute to efficient Met resynthesis from SAM through the SAM cycle.

Furthermore, SAMS interacts with several subunits of the vesicle complex (Fig. S4). These interactions suggest that SAMS may be actively transported via the vesicle-trafficking system in response to fruit ripening signals. Earlier studies revealed that SAMS localizes to the nucleus, cytosol, and plasma membrane^38,39^. Nuclear-localized SAMS may be responsible for the efficient production of SAM for subsequent DNA methylation^38^. Membrane-localized SAMS activity is suppressed via direct interaction with a FERONIA receptor-like kinase in response to environmental cues and hormones^39^. During the fruit-ripening stage, SAMS activity must be regulated to appropriately adjust the balance between methylation and ethylene production. The vesicle-trafficking system may be responsible for fine-tuning the subcellular distribution of SAMS to facilitate localized SAMS activity.

### Integrating networks and proteomics with a mathematical model

Network and system modeling can resolve many analytic problems in biology. A transcriptomics-based kinetic model of ethylene biosynthesis in the model fruit tomato has been constructed^28^, in which measured gene expression is used as an input. This model describes the basic metabolic flux and predicts mutational effects. The application of our approach, in which functional nodes were obtained through proteomics and the parameters were optimized on the basis of quantification results, was successfully demonstrated in research on fruit ripening in peach. In addition to previously established models based on known biochemical reactions, core nodes and regulatory processes in our model were identified via differential proteomics. These included the regulator IAA, which is specific to the regulation of peach fruit ripening, and a putative metabolon for Met synthesis and recycling. The regulatory impact of protein–protein interactions between MetE and SAHH on ethylene biosynthesis was predicted and simulated during peach fruit ripening. MetE therefore represents a novel regulator of ethylene biosynthesis.

In conclusion, differentially expressed proteins in peach fruit during ripening were screened with a peach mega mAb library. The identified proteins included important enzymes involved in the biosynthesis of ethylene and novel proteins correlated with fruit ripening. By combining antibody-based proteomics, transcriptomic data, and metabolite measurements, a mathematical ethylene biosynthesis model was constructed for peach fruit, and the function of key regulators in the network was validated and simulated.

## Materials and methods

### Plant materials and postharvest treatments

The SH cultivar CN16 and the MF cultivar CN13 were grown at the Zhengzhou Fruit Research Institute, Chinese Academy of Agriculture Sciences^40^. Samples of fruit were collected at S3, S4 I, S4 II, and S4 III in 2016. The end of the second exponential growth phase is stage S3, after which the fruit reaches its final size, and ripening begins in stage S4^41^. The ripening stages of MF CN13 fruit are based on the ethylene levels, in which S3 represents the end of the second exponential growth phase, when the fruit background color is green; in the S4 I substage, the fruit is prepared to release ethylene; in the S4 II substage, the fruit release little ethylene; and in the S4 III substage, the fruit release large amounts of ethylene, and fruit firmness begins to decline rapidly^24^. In contrast, the ripening stages of SH CN16 fruit are determined mainly according to fruit background color, in which the fruit background color is green with small amounts of white in the S4 I substage; the fruit background color is white with small amounts of green in S4 II; and the fruit background color is complete white in S4 III. After fruit collection, the mesocarps were dissected into small pieces, then immediately frozen in liquid nitrogen and stored at −80 °C until study.

### Measurement of ethylene production

Flesh firmness and ethylene production were measured according to protocols described previously^40^. The ethylene concentration was measured with a GC2010 gas chromatograph (Shimadzu, Kyoto, Japan) equipped with a flame ionization detector. The measurements were completed with three replicates for each sample, including 5–8 fruit per sample.

### Measurement of superoxide radical production (O_2_^−^)

The production rate of superoxide radicals (O_2_^−^) was measured as described previously^42^ with some modifications. Briefly, 0.5 g samples of peach fruit at different ripening stages were ground into powder with liquid nitrogen and extracted with 0.75 mL of 65 mM sodium phosphate buffer (pH 7.8). After centrifugation at 10,000 × *g* for 15 min, 10 μL of 10 mM hydroxylamine hydrochloride was added to 190 μL of the supernatant and mixed. One hour of incubation was performed at 37 °C, and then 100 μL of 58 mM sulphanilic acid and 100 μL of α-naphthyl amine were successively added to the mixture, which was allowed to react at 37 °C for 20 min. The absorbance was then measured at 530 nm for the qualification of O_2_^−^ production. The content of O_2_^−^ was calculated with a standard curve for NaNO_2_.

### Metabolite measurements

Freeze-dried peach fruit samples (*n* = 3) were crushed into powder, and 100 mg of the samples was extracted using 1.0 mL of 70% (v/v) aqueous methanol overnight at 4 °C. After centrifugation at 10,000 × *g* for 10 min, the extracts were absorbed (CNWBOND Carbon-GCB SPE Cartridge, 250 mg, 3 mL; ANPEL, Shanghai, China) and filtered (SCAA-104, 0.22 μm pore size; ANPEL, Shanghai, China) for LC-MS measurements.

Quantitative metabolomics data for the sample extracts were obtained using an LC-ESI-MS/MS system (HPLC, Shim-pack UFLC SHIMADZU CBM30A system; MS, Applied Biosystems 6500 Q TRAP). The experimental conditions were as follows: HPLC: column, Waters ACQUITY UPLC HSS T3 C18 (1.8 μm, 2.1 mm 100 mm); solvent system: water (0.04% acetic acid), acetonitrile (0.04% acetic acid); injection volume: 2 μL; gradient program: 100:0 V/V at 0 min, 5:95 V/V at 11.0 min, 5:95 V/V at 12.0 min, 95:5 V/V at 12.1 min, 95:5 V/V at 15.0 min; temperature: 40 °C; flow rate: 0.40 mL/min. The effluent was linked to an ESI-triple quadrupole-linear ion trap (Q TRAP)-MS system.

LIT and triple quadrupole (QQQ) scans were obtained from a Q TRAP, API 6500 TRAP LC/MS/MS system equipped with an ESI Turbo Ion-Spray interface. The system was operated in positive ion mode with Analyst 1.6 software (AB Sciex). The ESI source operation parameters were as follows: ion source and turbo spray; source temperature at 500 °C; ion spray voltage (IS) at 5500 V; ion source gas I (GSI), gas II (GSII), and curtain gas (CUR) were set at 55, 60, and 25.0 psi, respectively; and the collision gas (CAD) was set at high. Mass calibration and instrument tuning were conducted with 10 and 100 μmol/L polypropylene glycol solutions in 6500 Q TRAP and LIT modes, respectively. Overall, 6,500 Q TRAP scans were obtained as MRM experiments with the collision gas (nitrogen) set to 5 psi. DP and CE for individual MRM transitions were carried out with further optimization of DP and CE. A specific set of MRM transitions was closely monitored for each step based on the metabolites (Met, SAM, SAH, and Hcy) eluted within this step.

### Protein extraction

The mesocarps were ground to a fine powder after being frozen in liquid nitrogen in the presence of 2% polyvinylpolypyrrolidone. Two volumes of ice-cold extraction buffer [pH 8.0, 200 mM NaCl, 200 mM Tris-HCl, 50 mM sodium ascorbate 5 mM Na_4_EDTA, 1% NP-40, 1% Triton X-100, 1% Tween-20, 0.25% sodium metabisulfite, 10% glycerol, protease inhibitor cocktail (Roche, Basel, Switzerland), and 2 mM PMSF] were mixed with the ground materials. The materials were rotated for 2 h at 4 °C to lyse cells and then centrifuged at 15,000 × *g* for 30 min. The supernatants were filtered through a mesh (100 mm pores), and the protein abundance was measured with the bicinchoninic acid (BCA) assay^43^.

### Construction of the mAb library

Monoclonal antibodies were produced using a standard method^44^. First, the antigens were prepared with the mesocarp proteins extracted from stage S3–S4 III CN13 and CN16 fruit mixed with an equal volume of complete Freund’s adjuvant. BALB/c mice (*n* = 3) were immunized four times using an 60 μg of antigen initially, followed by the booster injections of 30 μg of antigen at 14-day intervals. For the second and subsequent immunizations, polyethylene glycol was used as the adjuvant. Hybridoma cells were generated by fusing isolated mouse spleen cells (2.0 × 10^7^/ml) and mouse P3X63Ag8.653 cells and then subcloned via limiting dilution. The verification of the hybridoma cell clones was performed using an enzyme-linked immunosorbent assay. Positive clones were then utilized in expansion cultures. Protein G was used to purify antibodies from culture medium supernatants.

### Antibody microarray screening

Each antibody was added (10 nl/spot) to the surface of nitrocellulose-coated microscope slides (FAST chip, CapitalBio, Beijing, China) using an Inkjet Microarrayer (Arrayjet Ltd., Roslin, UK) at 4 °C. The prepared slides were stored at −80 °C until use. For array screening, proteins (0.1 μg/μl) were labeled with NHS-biotin (Molecular Probes, Carlsbad, CA, USA).

The microarray slides were washed twice with TBS supplemented with 0.1% Tween 20 (TBS + 0.1% T_20_) and then blocked with 10% BSA in TBS + 0.1% T_20_ at 4 °C overnight. The slides were washed four times for 15 min each with TBS + 0.5% T_20_ and then incubated with 30 μg of biotin-labeled samples (30 ml final volume) at room temperature for 1 h. The slides were next washed four times with TBS + 0.5% T_20_ and then incubated with Cy3-labeled streptavidin (1:5000) at room temperature for 1 h. After four washes with TBS + 0.5% T_20_ and four washes with double-distilled H_2_O, the slides were centrifuged at 1500 rpm for 2 min to remove any residual buffer and dried. The dried slides were scanned with a GenePix microarray scanner (Axon Instruments, Union City, CA, USA). The Cy3 fluorescence signals were excited with a 532-nm laser and detected at 570 nm.

### Processing of mAbArray data

The Cy3 fluorescence intensities of the antibodies were quantified using the GenePix 6.0 program (Axon Instruments), and statistical analysis was carried out with the limma package (http://bioconductor.org/). The normalization of the data was performed via background correction and quantile normalization methods using the “background Correct” and “normalize Between Arrays” functions in the limma package, respectively. Unsupervised hierarchical clustering and principal component analysis (PCA) were used to assess the repeatability of the antibody microarray data from the three biological replicates. A linear model was applied to analyze the differentially expressed proteins in the antibody microarray with the “lmFit” and “eBayes” functions in the limma package. An antibody was considered significantly differentially expressed if an absolute fold-change of >1.5 and a *p*-value of <0.05 were observed.

Protein functional analysis was performed by first classifying the interacting proteins according to the peach genome annotations in MapMan (http://mapman.gabipd.org/)^45^. Functional enrichment analyses were completed via the hypergeometric test using R. Additionally, a PPI network was generated using the Cytoscape package (http://cytoscape.org/)^46^.

### WB analysis

SDS-PAGE was used to separate the proteins (20 μg) from each sample. The proteins were then transferred to polyvinylidene fluoride membranes, which were incubated with appropriate primary antibodies and an HRP-conjugated anti-mouse IgG secondary antibody. Immunoreactive bands were visualized with an enhanced chemiluminescence system (Biouniquer, China) and exposed to X-ray film (Kodak). Signal intensities were quantified by using the ImageJ program and normalized to the corresponding β-actin signal.

### Yeast two-hybrid assays

The full-length nucleotide sequences of SAHH and MetE were amplified and ligated into the Y2H bait and prey protein-expressing vectors pGADT7 and pGBKT7, respectively. The assay was performed according to the Matchmaker Gold Yeast Two-Hybrid System user manual (Clontech Laboratories, Inc., Japan). Briefly, the successfully sequenced bait and prey plasmids expressing candidate proteins were cotransformed into competent yeast cells via the LiAc method. The cultures were plated on DDO medium (SD-Leu/-Trp), and a single colony from each reaction was grown on QDO medium (SD-Ade/-His/-Leu/-Trp) and X-gal for the comparison of the interacting capacity.

### BIFC

MetE and SAHH were fused with the N- and C-termini of YFP, respectively. *Agrobacterium* was transformed with both plasmids carrying YFP^N^-MetE/ and YFP^C^-SAHH, and the cell suspensions were infiltrated into *Nicotiana benthamiana* leaves with syringes, followed by incubation for 3 days. Confocal microscopy was used to examine the leaves. The N-terminus of bZIP63 was selected as a positive control^47^, and YFP^N^-35S/ YFP^C^-35S was the negative control.

### IP and co-IP assay

Total peach protein samples (1 mg) were precleared with protein G resin (Invitrogen, Carlsbad, CA, USA) at 4 °C for 4 h and then incubated with a CNBr-conjugated peach antibody at 4 °C overnight. After extensive washes, the IP complexes were eluted with 0.2 M glycine (pH 2.5) and analyzed by SDS-PAGE followed by silver staining or WB analysis. Protein bands from the silver-stained gels corresponding to the band visualized in the WB were excised for in-gel trypsin digestion and MS analysis.

For co-IP experiments, proteins were incubated with the same antibodies used for the IP assays, with anti-mouse IgG as the control. After extensive washes, the IP complexes were eluted with 0.2 M glycine (pH 2.5) and subjected to in-solution trypsin digestion and MS analysis.

### LC-MS/MS analysis and data analysis

The trypsin-digested proteins were analyzed using a nanoACQUITY UPLC system (Waters Corporation) coupled to a Q Exactive Quadrupole-Orbitrap mass spectrometer (Thermo Fisher Scientific). A linear gradient from 2–40% ACN over 60 min was used for LC separation. Tandem mass spectra were processed with the PEAKS Studio program version 8.0 (Bioinfor Inc. CA). A PEAKS DB was set up to search the NCBI *Prunus persica* 201701 database (containing 59,075 entries), with trypsin as the digestion enzyme. Peptides showing a false discovery rate <0.01 were removed from the analysis. PEAKS Q was used to calculate peptide and protein abundances in the differential IP experiment. Data were normalized using the median peptide abundances. Proteins with an abundance fold-change >2.0 (*p* *<* 0.05; analysis of variance) were designated as differentially expressed proteins.

### RNA sequencing and data analysis

Total RNA was extracted from the mesocarps of CN13 and CN16 fruit harvested at stages S3, S4 I, S4 II, and S4 III as previously described^24^. RNA sequencing assays were carried out by Novogene Bioinformatics Technology Co., Ltd (Beijing, China). Differentially expressed genes were hierarchically clustered using the DESeq R package^48^. Additionally, a WGCNA was performed as previously described^23^. Abnormally expressed genes (FPKM < 1 or rowVars < 1) were excluded, and the expression levels of 14,421 genes were included in the WGCNA.

### Determination of biological age in the developmental stages of peach ripening

In agricultural applications, peach development is generally divided into the S1, S2, S3, and S4 stages. However, quantitative models require exact time points for calibration. In this work, we focused on developmental stages from S3 to S4III, and the biological ages of these different stages were determined by calculating the average time (days) from multiple peach trees during our measurements. Therefore, stages S3, S4I, S4II, and S4III started at 0 days, 12 days, 17 days, and 21 days after the S3 phase, respectively.

### Estimation of modeling parameters for quantification

Since the core enzymatic reactions of ethylene synthesis are conserved, for enzyme-catalyzed reactions, we adopted parameters from a study by Van de Poel et al.^28^. The mRNA and protein degradation rates obtained from the BioNumbers database were treated universally. Genetic/transcriptional regulation by transcription factors was estimated using the Hill function, and *ACO1* and *MetE* were transcriptionally regulated by their corresponding substrates in our model.

The parameters for the remaining biochemical reactions were optimized on the basis of available proteomic and transcriptomic data on protein abundance and mRNA fold-changes derived from experimental measurements [*YUC11*, *ACS1* mRNA fold-changes measured in;^24,40^ *MetE* and *ACO1* mRNA fold-changes measured in this study (Fig. 3)] and hormone production rates measured in the laboratory [IAA production rates at different stages were measured in^24^ in the same cultivar, CN13; ethylene production rates were obtained in this study]. The least squares minimization protocol in MATLAB was adopted to estimate those parameters. The goal of optimization is to minimize the summation of square errors (SSEs) for our experimental measurements. The differential equations of the full model are presented in Supplemental Notes S1 and S2 with all the parameters in Supplemental Table S2.

### Simulation details

MATLAB R2015b was used for numerically solving the differential equations. We used ODE45 to run the simulations. The initial concentrations of all relevant biomolecules were taken from both experimental measurements and BioNumbers databases or related work from other groups. A detailed list of the initial concentrations is provided in Supplemental Table S3.

## Supplementary information

SFigure S1

SFigure S2

SFigure S3

SFigure S4

SFigure S5

SFigure S6

STables

## Data Availability

The authors declare that the data supporting the study findings are presented in the article and Supplementary Information files or are available from the corresponding author upon request. The transcription data have been deposited at GenBank: accessions SRR5942182, SRR5942183, SRR5942180, SRR5942181, SRR5942186, SRR5942187, SRR5942184 and SRR5942185 (BioProject PRJNA398309).
