# Supplementary material for: Application of an antibody chip for screening differentially expressed proteins during peach ripening and identification of a metabolon in the SAM cycle to generate a peach ethylene biosynthesis model
Source: Hortic Res. 2020 Mar 15;7:31. doi: 10.1038/s41438-020-0249-9 (PMC7072073; doi:10.1038/s41438-020-0249-9)
Supplement: Supplementary file 1 — SFigure S1 [file 41438_2020_249_MOESM1_ESM.docx]

**
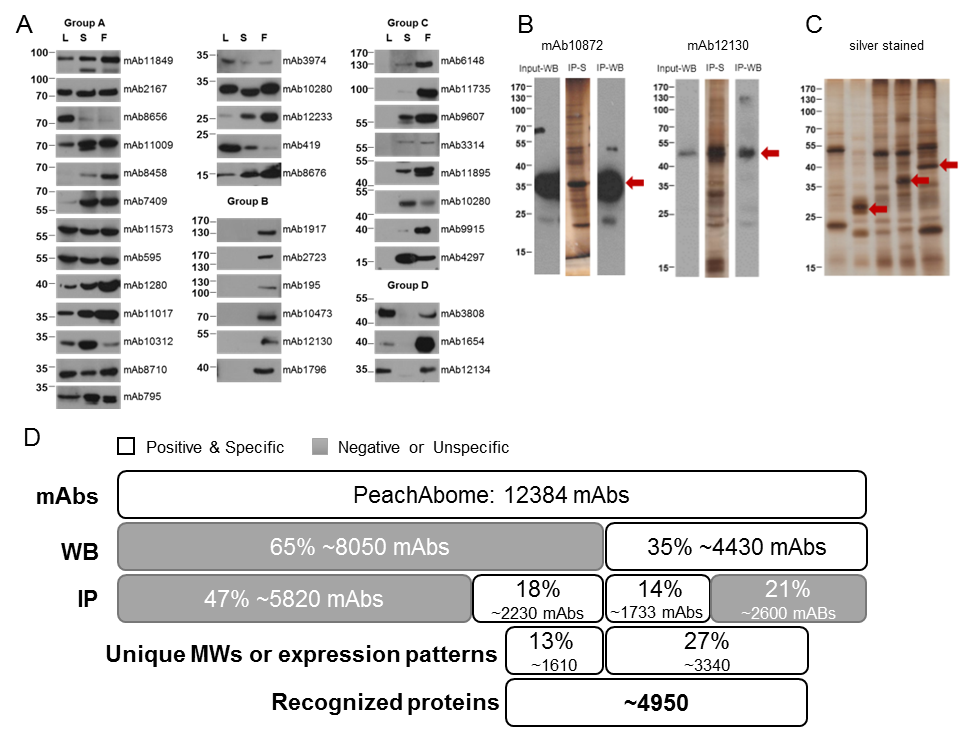
**

Fig. S1. Characterization of the peach mAb library. (a) Western blot (WB) analysis of the proteins derived from the leaves (L), seeds (S), and fruits (F) using 100 randomly selected antibodies. The mAbs were classified into several groups (Group a-d) according to the expression patterns in different tissues. The mAbs in Group A detect proteins expressed in leaves, seeds and fruits. The mAbs in Group B detect proteins expressed only in fruits. The mAbs in Group C and D detect proteins expressed in two of the tested tissues. (b) Representative IP assay results. The IP input was analysed by a WB and the IP output was analysed by silver staining and a WB. Arrows indicate the proteins specifically recognized by the antibodies. (c) Results of the IP assays with WB-negative antibodies. The IP output was analysed by silver staining. Arrows indicate the proteins recognized by the antibodies based on the criteria used to determine a successful IP. (d) Estimation of the library capacity. Among the 12384 antibodies, 35% antibodies (~4430) could specifically recognize proteins by WB which correspond to ~3340 different antigens according to the unique MW or expression pattern. About 40% (~1733) of WB positive mAbs could be used for IP. Among the 65% of WB negative mAbs, 27.7% (~2230) mAbs could be used in IP and result in ~1610 different antigens. By estimation, the mAb library can recognize ~4950 proteins. Numbers in the white boxes represent for the mAbs that could be applied for WB or IP, and numbers in the grey boxes represent for the mAbs that could not.
