# Supplementary material for: Application of an antibody chip for screening differentially expressed proteins during peach ripening and identification of a metabolon in the SAM cycle to generate a peach ethylene biosynthesis model
Source: Hortic Res. 2020 Mar 15;7:31. doi: 10.1038/s41438-020-0249-9 (PMC7072073; doi:10.1038/s41438-020-0249-9)
Supplement: Supplementary file 2 — SFigure S2 [file 41438_2020_249_MOESM2_ESM.docx]

**
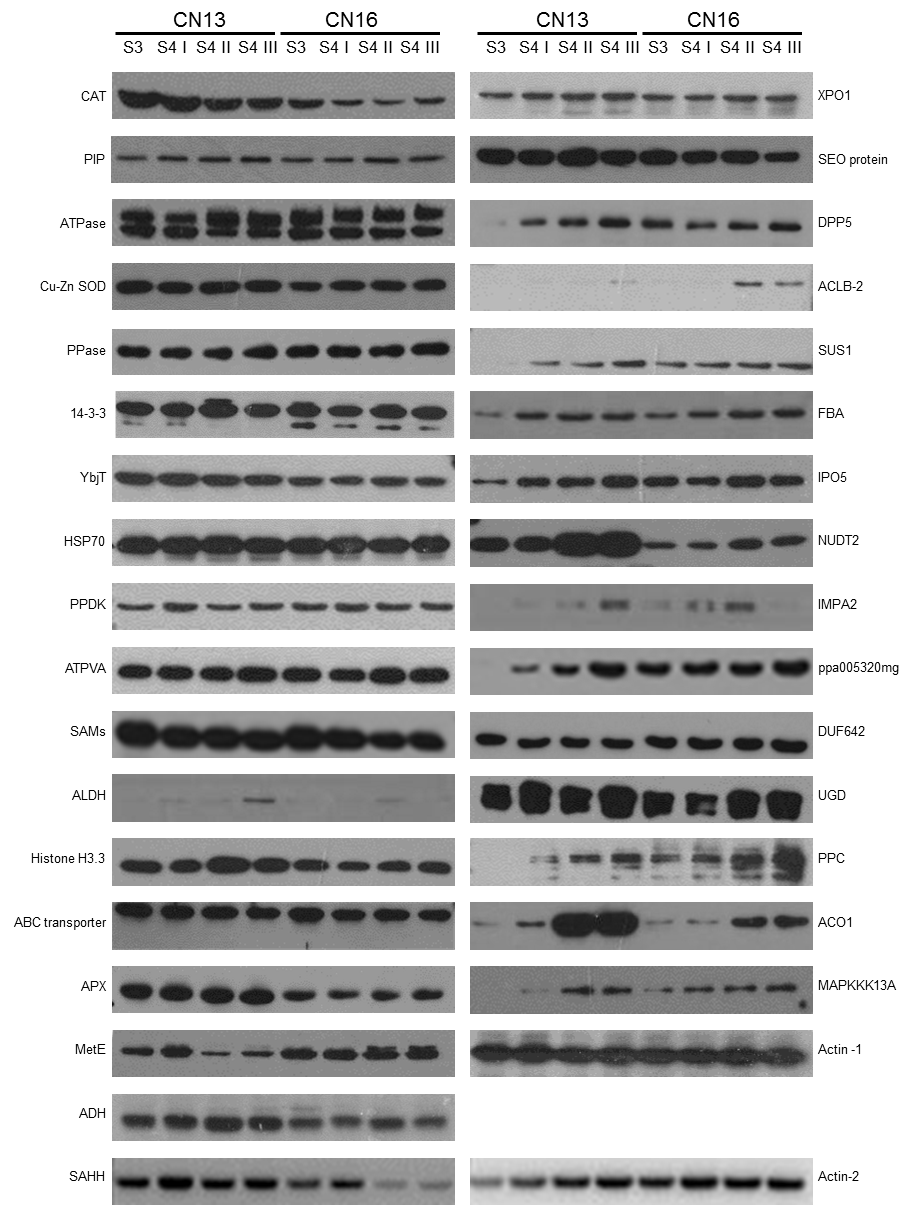
**

Fig. S2. Western blot analysis of the differently expressed proteins in CN13 and CN16 at S3-S4 III stages. Expression of the proteins related to peach ripening were analyzed by WB and compared between CN13 and CN16 at S3, S4 I, S4II and S4 III ripening stages. Actin-2 is used as internal control for WB of SAHH, and Actin-1 is used as internal control for the rest WB analyses.
