# Supplementary material for: Application of an antibody chip for screening differentially expressed proteins during peach ripening and identification of a metabolon in the SAM cycle to generate a peach ethylene biosynthesis model
Source: Hortic Res. 2020 Mar 15;7:31. doi: 10.1038/s41438-020-0249-9 (PMC7072073; doi:10.1038/s41438-020-0249-9)
Supplement: Supplementary file 3 — SFigure S3 [file 41438_2020_249_MOESM3_ESM.docx]

Fig. S3.


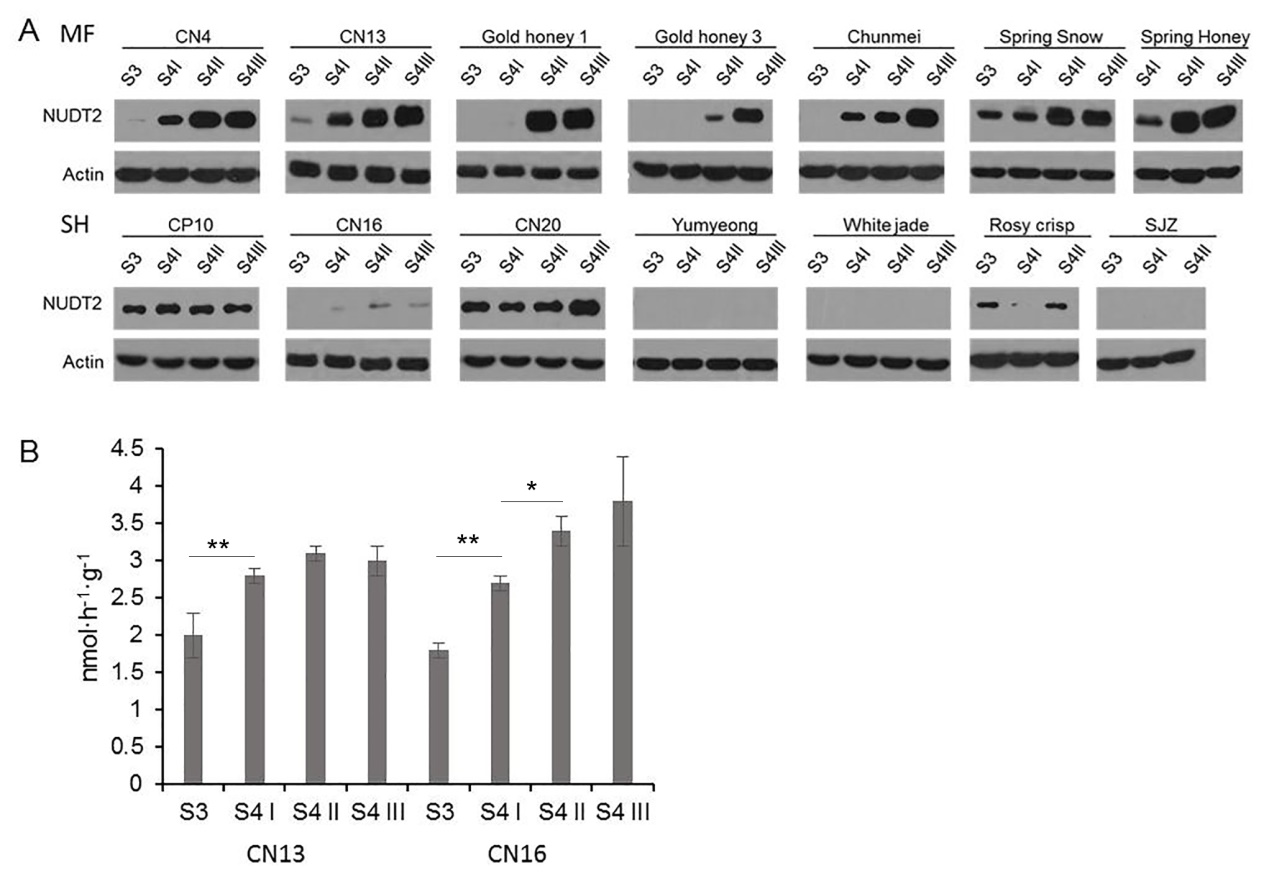


Fig. S3. Analysis of ROS contents in CN13 and CN16 during stages S3 and S4 I-III. The measurement was repeated with three biological replicates. Values are means ± SD, n=3. The significance of difference was analysed by student *t*-test. **, *p* < 0.01; *, *p* < 0.05.
