# Supplementary material for: Application of an antibody chip for screening differentially expressed proteins during peach ripening and identification of a metabolon in the SAM cycle to generate a peach ethylene biosynthesis model
Source: Hortic Res. 2020 Mar 15;7:31. doi: 10.1038/s41438-020-0249-9 (PMC7072073; doi:10.1038/s41438-020-0249-9)
Supplement: Supplementary file 4 — SFigure S4 [file 41438_2020_249_MOESM4_ESM.docx]

**
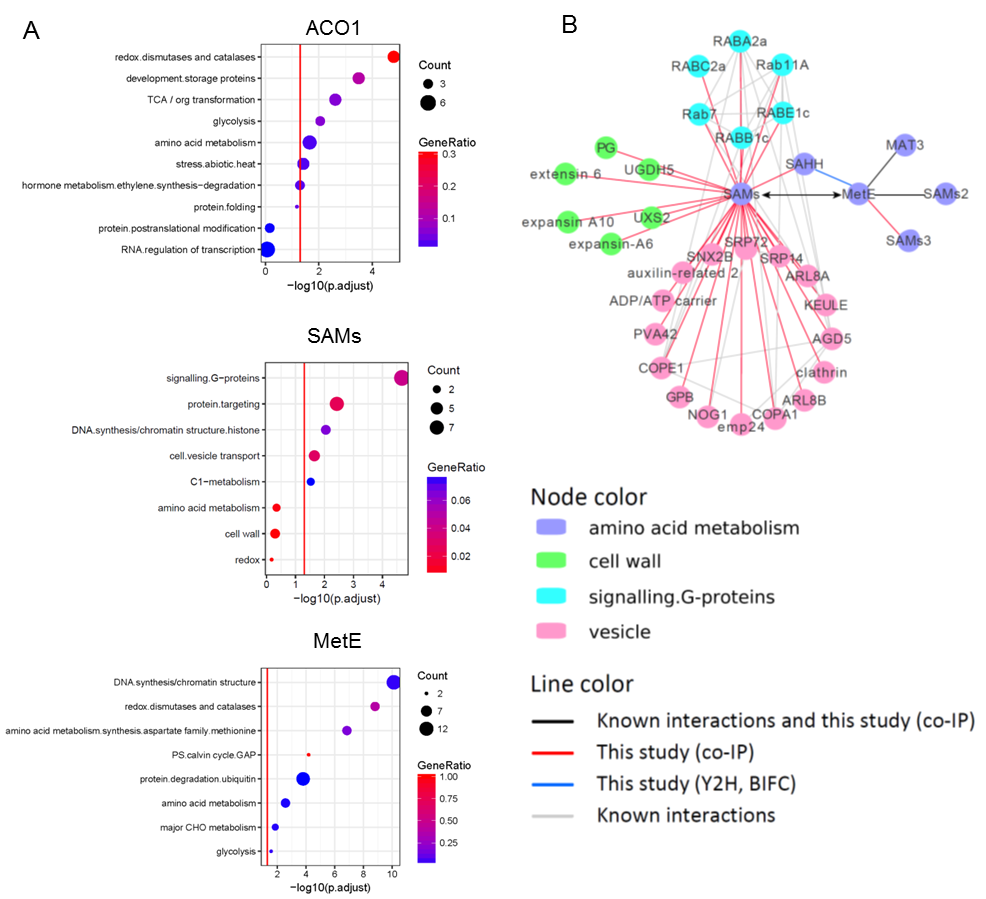
**

Fig. S4. Co-IP analysis of proteins interact with ACO1, MetE and SAMs. (a) The proteins that interact with ACO1, MetE and SAMs were co-immuno precipitated with anti-ACO1, anti-MetE and anti-SAMs mAbs, respectively and identified by MS. The interactive proteins were functionally classified by Mapman software. The enriched categories of interactive protein for each bait protein are shown. The bubble size indicates the number of proteins included in each category. Different colors correspond to the ratio of genes in each category. The vertical red line indicates the threshold of significance (*p* < 0.05). (b) MetE and SAMs protein-protein interaction network at S4 III stage in MF and SH peach fruit. The interactive proteins related to G-protein signalling, cell vesicle transport, protein targeting, and cell wall synthesis and modification and amino acid metabolism are presented. Double-headed-arrow represent for interaction identified in reciprocal co-IP assays.
