# Supplementary material for: Application of an antibody chip for screening differentially expressed proteins during peach ripening and identification of a metabolon in the SAM cycle to generate a peach ethylene biosynthesis model
Source: Hortic Res. 2020 Mar 15;7:31. doi: 10.1038/s41438-020-0249-9 (PMC7072073; doi:10.1038/s41438-020-0249-9)
Supplement: Supplementary file 5 — SFigure S5 [file 41438_2020_249_MOESM5_ESM.docx]

**
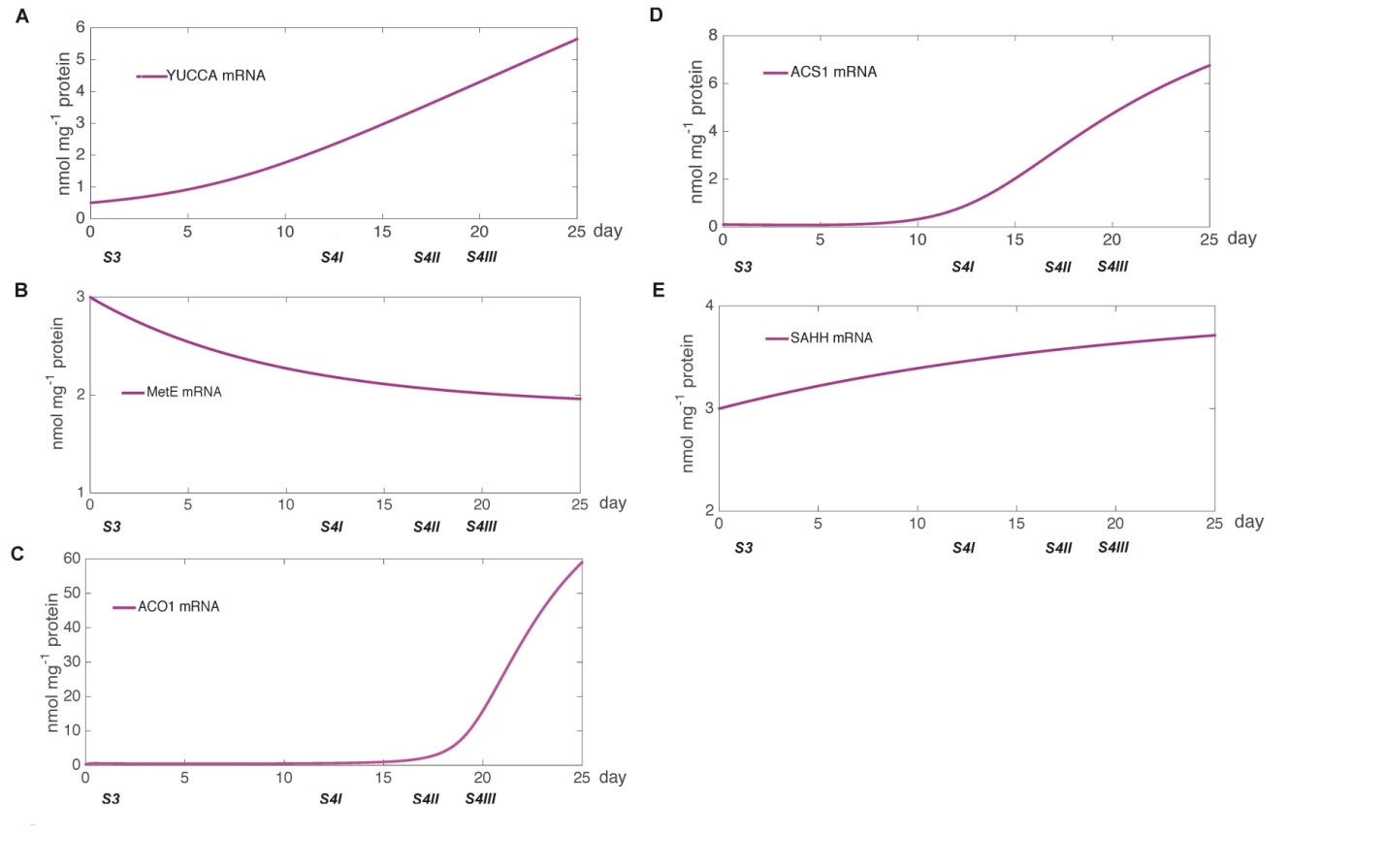
**

Fig. S5. Dynamics of mRNA dynamics expression during fruit ripening in peach. Expression of *YUCCA* (A), *MetE* (B), *ACO1* (C), *ACS1* (D), and *SAHH* (E) were compared with previous publications (Pan et al., 2015) or transcriptome results at indicated stages.
