# Supplementary material for: Application of an antibody chip for screening differentially expressed proteins during peach ripening and identification of a metabolon in the SAM cycle to generate a peach ethylene biosynthesis model
Source: Hortic Res. 2020 Mar 15;7:31. doi: 10.1038/s41438-020-0249-9 (PMC7072073; doi:10.1038/s41438-020-0249-9)
Supplement: Supplementary file 6 — SFigure S6 [file 41438_2020_249_MOESM6_ESM.docx]

**
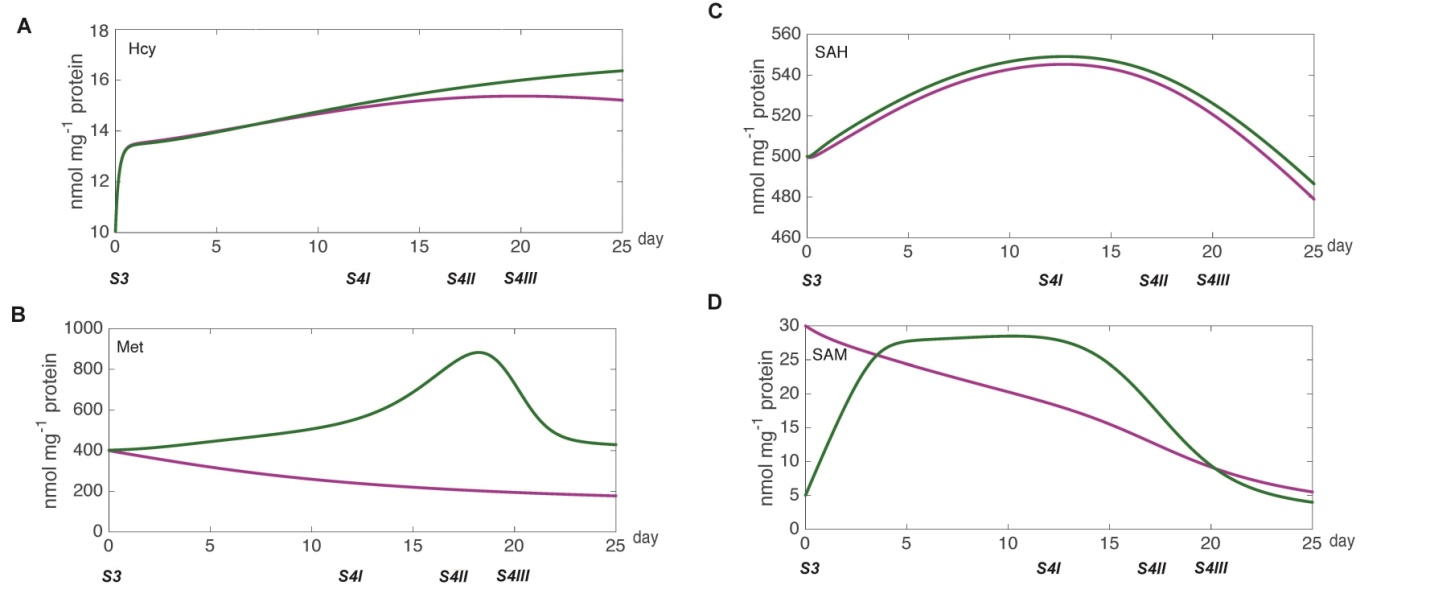
**

Fig. S6. Metabolite dynamics of peach fruit during ripening calculated with systemic biological model. Metabolite concentration changes are in general correlated with experimental measurements.
