# Supplementary material for: Application of an antibody chip for screening differentially expressed proteins during peach ripening and identification of a metabolon in the SAM cycle to generate a peach ethylene biosynthesis model
Source: Hortic Res. 2020 Mar 15;7:31. doi: 10.1038/s41438-020-0249-9 (PMC7072073; doi:10.1038/s41438-020-0249-9)
Supplement: Supplementary file 7 — STables [file 41438_2020_249_MOESM7_ESM.docx]

Table S1. IP-LC_MS/MS identification of antigens related with peach ripening.

Δ stage, comparation of protein expression between different ripening stages; Δ (MF-SH), comparation of protein expression in at least one stage between MF and SH cultivars; Up, monotonically increased protein; Down, monotonically decreased protein; -, no significant differences.

| mAb No. | Accession GI | Protein Name | MW (Dal) | Δ stage | Δ (MF-SH) |
| --- | --- | --- | --- | --- | --- |
| mAb9578 | gi\|595857598 | Catalase, CAT | 57071 | Down | - |
| mAb11281 | gi\|595857598 | Catalase, CAT | 57071 | Down | - |
| mAb10695 | gi\|595857598 | Catalase, CAT | 57071 | Down | - |
| mAb11098 | gi\|595857598 | Catalase, CAT | 57071 | Down | - |
| mAb12095 | gi\|403234152 | aquaporin, PIP | 30899 | Down | Down |
| mAb11095 | gi\|462400619 | Chromosome segregation ATPase, ATPase | 70236 | - | - |
| mAb11630 | gi\|400532665 | Cu-Zn SOD | 15380 | - | - |
| mAb12288 | gi\|462401473 | Pyrophosphatase, PPase | 26181 | - | Up |
| mAb11631 | gi\|595946001 | 14-3-3 | 28098 | - | Up |
| mAb11986 | gi\|595797200 | Uncharacterized conserved protein YbjT | 37181 | - | - |
| mAb739 | gi\|462402800 | heat shock 70 kDa protein, HSP70 | 69578 | - | - |
| mAb11935 | gi\|596285319 | Pyruvate phosphate dikinase, PPDK | 130674 | - | Up |
| mAb12174 | gi\|15982954 | V-type H^+^-transporting ATPase subunit A, ATPVA | 68690 | - | - |
| mAb6204 | gi\|452090878 | S-adenosylmethionine synthase, SAMs | 42909 | Down | - |
| mAb595 | gi\|595802986 | Aldehyde dehydrogenase, ALDH | 54283 | - | - |
| mAb12016 | gi\|529279911 | histone varient H3.3 | 15406 | - | - |
| mAb791 | gi\|596298453 | ABC transpoter | 40509 | - | - |
| mAb10943 | gi\|595828935 | L-ascorbate peroxidase, APX | 27367 | - | - |
| mAb10717 | gi\|595828935 | L-ascorbate peroxidase, APX | 27367 | - | - |
| mAb4414 | gi\|462399797 | 5-methyltetrahydropteroyltriglutamate-homocysteine methyltransferase, MetE | 84669 | Down | Down |
| mAb11317 | gi\|595792363 | class III alcohol dehydrogenase, ADH | 44694 | - | Up |
| mAb726 | gi\|596055759 | protein EXPORTIN 1A, XPO1 | 123576 | Up | Up |
| mAb11894 | gi\|462422145 | SEO protein | 82913 | - | Up |
| mAb11849 | gi\|462422100 | dipeptidyl-peptidase 5, DPP5 | 75927 |  |  |
| mAb11920 | gi\|596127635 | ATP-citrate synthase beta chain protein 2, ACLB-2 | 65912 | Up | - |
| mAb9164 | gi\|385282642 | sucrose synthase 1, SUS1 | 92619 | Up | Up |
| mAb10758 | gi\|385282642 | sucrose synthase 1, SUS1 | 92619 | Up | Up |
| mAb12161 | gi\|385282642 | sucrose synthase 1, SUS1 | 92619 | Up | Up |
| mAb11239 | gi\|595805110 | fructose-bisphosphate aldolase, FBA | 38413 | - | - |
| mAb11247 | gi\|595805110 | fructose-bisphosphate aldolase, FBA | 38413 | - | - |
| mAb10876 | gi\|595822812 | Importin-5, IPO5 | 123445 | Up | - |
| mAb10960 | gi\|595937557 | Nudix hydrolase 2, NUDT2 | 39997 | Up | - |
| mAb12120 | gi\|596141830 | importin subunit alpha-2, IMPA2 | 58301 | - | - |
| mAb585 | gi\|595931518 | hypothetical protein PRUPE_ppa005320mg | 50833 | - | - |
| mAb10896 | gi\|596001390 | DUF642 | 39660 | - | - |
| mAb11102 | gi\|464896000 | UDP-D-glucose dehydrogenase, UGD | 53000 | - | - |
| mAb11893 | gi\|464896000 | UDP-D-glucose dehydrogenase, UGD | 53000 | - | - |
| mAb11336 | gi\|462409547 | Phosphoenolpyruvate carboxylase, PPC | 110063 | Up | - |
| mAb10786 | gi\|16588828 | 1-aminocyclopropane-1-carboxylate oxidase 1, ACO1 | 36172 | Up | Up |
| mAb10785 | gi\|16588828 | 1-aminocyclopropane-1-carboxylate oxidase 1, ACO1 | 36172 | Up | Up |
| mAb10712 | gi\|595895735 | Mitogen-activated protein kinase kinase kinase 13A, MAPKKK13A | 147377 | Up | - |
| mAb12067 | gi\|596144191 | S-adenosyl-L-homocysteine hydrolase, SAHH | 53361 | Down | Down |

**Table S2.** The parameters used in the mathematic model. Estimated parameters and their units are listed.

| **Function** | **Abbreviation** | **Values** | **Unit** | **Data Source** |
| --- | --- | --- | --- | --- |
| ACO1 mRNA production | Gaco1e | 15.00 | nmol mg protein^-1^ d^-1^ | Optimization |
| ACS1 mRNA production | Gacs1e | 1.00 | nmol mg protein^-1^ d^-1^ | Optimization |
| ACS2 mRNA production | Gacs2e | 0.20 | nmol mg protein^-1^ d^-1^ | Optimization |
| ACS6 mRNA production | Gacs6e | 0.05 | nmol mg protein^-1^ d^-1^ | Optimization |
| MetE mRNA production | Gmetee | 0.30 | nmol mg protein^-1^ d^-1^ | Optimization |
| YUCCA mRNA production | Gyuccae | 0.30 | nmol mg protein^-1^ d^-1^ | Optimization |
| SAHH mRNA production | Gsahhe | 0.20 | nmol mg protein^-1^ d^-1^ | Optimization |
| ACO1 mRNA degradation | kedaco1e | 0.10 | d^-1^ | Database |
| ACS1 mRNA degradation | kedacs1e | 0.10 | d^-1^ | Database |
| ACS2 mRNA degradation | Kedacs2e | 0.10 | d^-1^ | Database |
| ACS6 mRNA degradation | Kedacs6e | 0.10 | d^-1^ | Database |
| MetE mRNA degradation | kedmetee | 0.10 | d^-1^ | Database |
| YUCCA mRNA degradation | kedyuccae | 0.10 | d^-1^ | Database |
| SAHH mRNA degradation | kedsahhe | 0.10 | d^-1^ | Database |
| ACO1 Protein production rate from mRNA | Ktaco | 0.22 | nmol d^-1^ mg protein^-1^ d^-1^ nmol mRNA^-1^ | Van de Poel et al. 2014 |
| ACS1 Protein production from mRNA | ktacs | 0.028 | nmol d^-1^ mg protein^-1^ d^-1^ nmol mRNA^-1^ | Van de Poel et al. 2014 |
| ACS2 Protein Production from mRNA | ktacs | 0.028 | nmol d^-1^ mg protein^-1^ d^-1^ nmol mRNA^-1^ | Van de Poel et al. 2014 |
| ACS6 Protein production from mRNA | ktacs | 0.028 | nmol d^-1^ mg protein^-1^ d^-1^ nmol mRNA^-1^ | Van de Poel et al. 2014 |
| MetE Protein production from mRNA | ktmete | 0.2 | nmol d^-1^ mg protein^-1^ d^-1^ nmol mRNA^-1^ | Optimization |
| YUCCA Protein production from mRNA | ktyucca | 0.8 | nmol d^-1^ mg protein^-1^ d^-1^ nmol mRNA^-1^ | Optimization |
| SAHH protein production from mRNA | ktsahh | 0.2 | nmol d^-1^ mg protein^-1^ d^-1^ nmol mRNA^-1^ | Optimization |
| ACO1 Protein degradation | kpdaco1 | 0.2 | d^-1^ | Database |
| ACS1 Protein degradation | kpdacs1 | 0.2 | d^-1^ | Database |
| ACS2 Protein degradation | kpdacs2 | 0.2 | d^-1^ | Database |
| ACS6 Protein degradation | kpdacs6 | 0.2 | d^-1^ | Database |
| MetE Protein degradation | kpdmete | 0.2 | d^-1^ | Database |
| YUCCA Protein degradation | kpdyucca | 0.5 | d^-1^ | Database |
| SAHH protein degradation | kpdsahh | 0.3 | d^-1^ | Optimization |
| Ethylene Synthesis | kaco | 0.2 | mg protein h nmol^-1^ d^-1^ | Van de Poel et al. 2014 |
| ACC Synthesis | kacs1 | 2.2 | mg protein h nmol^-1^ d^-1^ | Van de Poel et al. 2014 |
| ACC Synthesis | kacs2 | 0.13 | mg protein h nmol^-1^ d^-1^ | Van de Poel et al. 2014 |
| ACC Synthesis | kacs6 | 0.016 | mg protein h nmol^-1^ d^-1^ | Van de Poel et al. 2014 |
| L-Met from HCY | kmete | 0.2 | mg protein h nmol^-1^ d^-1^ | Database |
| IAA production from IPA | kiaa | 0.2 | mg protein h nmol^-1^ d^-1^ | Database |
| L-Met to SAM | ksams | 0.0004 | mg protein h nmol^-1^ d^-1^ | Database |
| SAH to HCY | ksahh | 0.2 | mg protein h nmol^-1^ d^-1^ | Optimization |
| Ethylene diffusivity | kdiff | 4.4 | h^-1^ | Van de Poel et al. 2014 |

## Unit definition: 1g peach usually generate ~ 1.0 mg protein after protein extraction. Therefore the total protein concentration is 1.0 mg/g peach, which is ~ 1.0 mg/mL (1 g/L) when treated the density as around water.

**Table S3.** The initial concentrations of molecular species used in the mathematic model.

| **Biomolecular Species** | **Initial Values** | | **Unit** | **Data Source** |
| --- | --- | --- | --- | --- |
| ACO1 mRNA | | 0.1 | nmol | Measurement |
| ACS1 mRNA | | 0.1 | nmol | Measurement |
| ACS2 mRNA | | 0.1 | nmol | Measurement |
| ACS6 mRNA | | 0.1 | nmol | Measurement |
| MetE mRNA | | 3.0 | nmol | Measurement |
| SAHH mRNA | | 3.0 | nmol | Measurement |
| YUCCA mRNA | | 0.5 | nmol | Pan et al |
| ACO1 Protein | | 1.5 | nmol | Measurement |
| ACS1 Protein | | 0.01 | nmol | Database |
| ACS2 Protein | | 0.01 | nmol | Database |
| ACS6 Protein | | 0.01 | nmol | Database |
| MetE Protein | | 3.0 | nmol | Measurement |
| YUCCA Protein | | 0.2 | nmol | Pan et al |
| SAHH mRNA | | 3.0 | nmol | Measurement |
| ACC | | 0.1 | Ng/g | Poel et al |
| SAM | | 5.0 | Ng/g | Poel et al |
| HCY | | 12.0 | Ng/g | Bio-number |
| IAA | | 1.0 | Ng/g | Measurement |
| Ethylene | | 0.5 | Ng/g | Measurement |
| SAH | | 500 | Ng/g | Measurement |
| SAM | | 3000 | Ng/g | Measurement |
| Methionine | | 400 | Ng/g | Measurement |

**Notes S1.** Comparisons of mRNA expressions from simulations with RNA-Seq data after model calibration.

Part of our parameters on gene expression and protein production are optimized from experimental measurements. Pan et al. (Pan et al., 2015) showed that *YUC11* gene was up-regulated by ~5 fold in peach, while *ACS1* gene was highly up-regulated ~ 60 fold. Additionally, our OMICS data show that *MetE* expression will be down-regulated by ~50%, while *ACO1* expression is up-regulated by around 80 fold. These parameters are used in optimizing the related-parameters for gene expressions. After parameter estimation, the simulated changes of these mRNAs are also shown as below.

**Notes S2.** Ordinary differential equations of the modeled system (parameters in Supplemental Table S2, and see method in main text for the model construction).

*d(ACO1e)/dt = Gaco1e*****H****(ETHint, N_ETHACO1e_, λ_ETHACO1e_) - kedaco1e*ACO1e*

*d(ACS1e)/dt = Gacs1e*****H****(IAA, N_IAAACS1e_, λ_IAAACS1e_) - kedacs1e*ACS1e*

*d(ACS2e)/dt = Gacs2e*****H****(IAA, N_IAAACS2e_,* λ*_IAAACS2e_) - kedacs2e*ACS2e*

*d(ACS6e)/dt = Gacs6e*****H****(IAA, N_IAAACS6e_, λ_IAAACS6e_) - kedacs6e*ACS6e*

*d(METEe)/dt = Gmetee*****H****(HCY, N_HCYMETEe_, λ_HCYMETEe_) - kedmetee*METEe*

*d(YUCCAe)/dt = Gyuccae - kedyuccae*YUCCAe*

*d(SAHHe)/dt = Gsahhe - kedsahhe*SAHHe*

*d(ACO1)/dt = ktaco*ACO1e - kpdaco1*ACO1*

*d(ACS1)/dt = ktacs*ACS1e - kpdacs1*ACS1*

*d(ACS2)/dt = ktacs*ACS2e - kpdacs4*ACS2*

*d(ACS6)/dt = ktacs*ACS6e - kpdacs6*ACS6*

*d(METE)/dt = ktmete*METEe - kpdmete*METE*

*d(YUCCA)/dt = ktyucca*YUCCAe - kpdyucca*YUCCA*

*d(SAHH)/dt = ktsahh*SAHHe - kpdsahh*SAHH*

*d(SAM)/dt = ksams*SAMS*Met - kacs2*SAM*ACS2 - kacs4*SAM*ACS4 - kacs6*SAM*ACS6-****H****(HCY, N_SAHinhibit_, λ_SAHinhibit_)*SAM*kenzyme*

*d(ACC)/dt = kacs1*SAM*ACS1 + kacs2*SAM*ACS2 + kacs6*SAM*ACS6 - kaco1*ACC*ACO1*

*d(HCY)/dt =* ***H****(HCY, N_HCYinhibit_, λ_HCYinhibit_)*SAHH*SAH*ksahh - METE*HCY*kmete*SAHH*

*dSAHdt =* ***H****(HCY, N_SAHinhibit_, λ_SAHinhibit_)*SAM*kenzyme -* ***H****(HCY, N_HCYinhibit_, λ_HCYinhibit_)*SAHH*SAH*ksahh*

*d(ETHint)/dt = kaco1*ACC*ACO1 - kdiff*(ETHint-ETHout)*

*d(IAA)/dt = ktiaa*YUCCA*

*d(MET)/dt = - ksams*SAMS*MET + METE*HCY*kmete*SAHH- koff*MET*
